# Supplementary material for: Polyketide synthase gene clusters in the ericoid mycorrhizal fungus Oidiodendron maius: genome-wide prediction, expression profiling, and functional characterization of knockout mutants
Source: Front Plant Sci. 2026 Sep 16;17:1924430. doi: 10.3389/fpls.2026.1924430 (PMC13623903; doi:10.3389/fpls.2026.1924430)
Supplement: Supplementary file 1 [file SupplementaryFile1.zip › Supplementary Materials/Supplementary Folder 1.zip!/Oidma1_AssemblyScaffolds_Repeatmasked/knownclusterblast/cluster58/jgi.p_Oidma1_173820_mibig_hits.html]

| MIBiG Protein | Description | MIBiG Cluster | MiBiG Product | % ID | % Coverage | BLAST Score | E-value |
| --- | --- | --- | --- | --- | --- | --- | --- |
| AMM63163.1 | AniL | BGC0001371 | Echinocandin\_B\_biosynthetic\_gene\_cluster | 35 | 102.875175316 | 865 | 7.6e-251 |
| OAQ83754.1 | ABC\_multidrug\_transporter | BGC0001358 | Leucinostatins\_biosynthetic\_gene\_cluster | 36 | 107.854137447 | 862 | 8.4e-250 |
| EPE34342.1 | P-loop\_containing\_nucleoside\_triphosphate\_hydrolase | BGC0001035 | Pneumocandin\_biosynthetic\_gene\_cluster | 36 | 92.2159887798 | 795 | 9.6e-230 |
| ACB12564.1 | Fum19 | BGC0000063 | Fumonisin\_biosynthetic\_gene\_cluster | 33 | 107.433380084 | 715 | 9.7e-206 |
| AAN74822.1 | Fum19p | BGC0000062 | Fumonisin\_biosynthetic\_gene\_cluster | 33 | 106.732117812 | 695 | 1.8e-199 |
| AMR44284.1 | ABC\_transporter | BGC0001398 | Phomopsins\_biosynthetic\_gene\_cluster | 32 | 90.603085554 | 537 | 4.7e-152 |
| AFT91383.1 | EcdL | BGC0000338 | Echinocandin\_B\_biosynthetic\_gene\_cluster | 32 | 65.0771388499 | 441 | 4.6e-123 |
| CRG85577.1 | hypothetical\_protein | BGC0001402 | Cyclochlorotine\_biosynthetic\_gene\_cluster | 27 | 48.0364656381 | 239 | 2e-62 |
| AAC44070.1 | unknown | BGC0000797 | Sphingan\_polysaccharide\_biosynthetic\_gene\_cluster | 28 | 34.6423562412 | 154 | 8.6e-37 |
| AAP57696.1 | putative\_secretion\_protein | BGC0000768 | Gellan\_polysaccharide\_biosynthetic\_gene\_cluster | 29 | 34.6423562412 | 154 | 1.1e-36 |
| CAI47637.1 | putative\_ABC-type\_aminoglycoside\_exporter | BGC0000692 | Apramycin\_biosynthetic\_gene\_cluster | 27 | 34.1514726508 | 154 | 1.1e-36 |
| CAG34715.1 | putative\_ABC\_transporter | BGC0000713 | Ribostamycin\_biosynthetic\_gene\_cluster | 26 | 35.2033660589 | 148 | 6.2e-35 |
| CAF31838.1 | putative\_hygromycin\_B\_exporter | BGC0000699 | Hygromycin\_B\_biosynthetic\_gene\_cluster | 26 | 40.7433380084 | 146 | 1.8e-34 |
| ABW74893.1 | ABC\_transporter | BGC0000759 | Diutan\_polysaccharide\_biosynthetic\_gene\_cluster | 27 | 34.4319775596 | 146 | 3.1e-34 |
| AHH99931.1 | ABC\_transporter\_protein | BGC0000002 | Aculeximycin\_biosynthetic\_gene\_cluster | 30 | 21.9495091164 | 143 | 1.5e-33 |
| EDY04298.1 | ABC\_transporter-related\_protein | BGC0000516 | Geobacillin\_II\_biosynthetic\_gene\_cluster | 28 | 37.0266479663 | 143 | 2e-33 |
| CAG38700.1 | putative\_ABC-transporter,\_ATP-binding\_protein | BGC0000708 | Lividomycin\_biosynthetic\_gene\_cluster | 28 | 35.2734922861 | 143 | 2e-33 |
| AAX31531.1 | putative\_ABC\_transporter | BGC0000336 | Daptomycin\_biosynthetic\_gene\_cluster | 24 | 36.6760168303 | 141 | 9.9e-33 |
| CAJ88629.1 | putative\_netropsin\_resistance\_protein\_subunit\_2 | BGC0000327 | Congocidine\_biosynthetic\_gene\_cluster | 26 | 34.3618513324 | 139 | 3.8e-32 |
| CAD89782.1 | hypothetical\_protein | BGC0001010 | Melithiazol\_biosynthetic\_gene\_cluster | 26 | 38.0084151473 | 138 | 8.4e-32 |
| AIS24845.1 | Dst21 | BGC0001147 | Distamycin\_biosynthetic\_gene\_cluster | 25 | 34.2917251052 | 132 | 3.5e-30 |
| BAL15734.1 | ABC\_transporter | BGC0000432 | Streptothricin\_biosynthetic\_gene\_cluster | 27 | 34.3618513324 | 131 | 7.8e-30 |
| ALX37944.1 | GaeT | BGC0001388 | Gassericin\_E\_biosynthetic\_gene\_cluster | 25 | 40.6732117812 | 128 | 5.1e-29 |
| BAM09402.1 | putative\_ABC-transporter | BGC0000619 | Gassericin\_T\_biosynthetic\_gene\_cluster | 25 | 40.6732117812 | 128 | 5.1e-29 |
| CBL93714.1 | ABC\_multidrug\_transporter | BGC0000360 | GE81112\_biosynthetic\_gene\_cluster | 26 | 33.7307152875 | 128 | 8.7e-29 |
| AAK73498.1 | AmphG | BGC0000015 | Amphotericin\_biosynthetic\_gene\_cluster | 25 | 35.553997195 | 126 | 1.9e-28 |
| AET14838.1 | multidrug\_resistance\_protein | BGC0001274\_c2 | Non-acetylated\_open-chain\_sophorolipid\_/\_diacetylated\_lactonic | 26 | 38.920056101 | 126 | 3.3e-28 |
| ADI10107.1 | cytolysin\_B\_transport\_protein | BGC0001229 | SBI\_06990\_alpha\_/\_SBI\_06989\_beta\_biosynthetic\_gene\_cluster | 26 | 35.4137447405 | 122 | 3.6e-27 |
| CAC20925.1 | PimB\_protein | BGC0000125 | Pimaricin\_biosynthetic\_gene\_cluster | 26 | 39.6914446003 | 122 | 4.8e-27 |
| XP\_011392729.1 | ABC\_transporter | BGC0001281 | Ustilagic\_acid\_biosynthetic\_gene\_cluster | 27 | 33.3800841515 | 121 | 6.2e-27 |
| ADC96668.1 | ABC\_transporter | BGC0001076 | Liposidomycin\_biosynthetic\_gene\_cluster | 25 | 34.221598878 | 120 | 1.4e-26 |
| CAE53357.1 | ABC\_transporter | BGC0000440 | Teicoplanin\_biosynthetic\_gene\_cluster | 25 | 37.7980364656 | 119 | 3.1e-26 |
| CAG15016.1 | ABC\_transporter | BGC0000441 | Teicoplanin\_biosynthetic\_gene\_cluster | 25 | 37.7980364656 | 119 | 3.1e-26 |
| EYT83450.1 | ABC\_transporter | BGC0001213 | Nataxazole\_biosynthetic\_gene\_cluster | 25 | 39.5511921459 | 118 | 6.9e-26 |
| ABL74935.1 | TlmT | BGC0001048 | Tallysomycin\_biosynthetic\_gene\_cluster | 26 | 36.0448807854 | 116 | 2e-25 |
| WP\_006126653.1 | ABC\_transporter | BGC0000554 | SRO15-3108\_biosynthetic\_gene\_cluster | 26 | 34.9228611501 | 116 | 3.4e-25 |
| ANP43732.1 | ThsT | BGC0001391 | Thusin\_biosynthetic\_gene\_cluster | 25 | 35.0631136045 | 115 | 5.8e-25 |
| BAJ05895.1 | putative\_ABC\_multidrug\_resistance\_transporter | BGC0000872 | A-90289\_biosynthetic\_gene\_cluster | 25 | 34.0112201964 | 114 | 7.6e-25 |
| AHF21240.1 | CrnT | BGC0000500 | Carnolysin\_A1\_/\_carnolysin\_A2\_biosynthetic\_gene\_cluster | 22 | 34.5021037868 | 114 | 1.3e-24 |
| BAB04170.1 | lantibiotic\_mersacidin\_transporter\_system | BGC0000517 | Haloduracin\_alpha\_/\_haloduracin\_beta\_biosynthetic\_gene\_cluster | 25 | 33.5203366059 | 113 | 2.2e-24 |
| ABV83219.1 | CppH | BGC0000116 | Nystatin-like\_Pseudonocardia\_polyene\_biosynthetic\_gene\_cluster | 26 | 26.2272089762 | 112 | 4.9e-24 |
| AAK81823.1 | ABC\_transporter | BGC0000326 | Complestatin\_biosynthetic\_gene\_cluster | 26 | 35.4838709677 | 111 | 6.4e-24 |
| CCC55903.1 | putative\_ABC\_transporter,\_ATPase\_and\_permease\_component | BGC0000973 | Collismycin\_A\_biosynthetic\_gene\_cluster | 26 | 30.7854137447 | 111 | 1.1e-23 |
| ABD65953.1 | ABC\_transporter | BGC0000341 | Enduracidin\_biosynthetic\_gene\_cluster | 24 | 39.6914446003 | 111 | 1.1e-23 |
| ACQ63630.1 | ABC\_multidrug\_resistance\_transporter | BGC0000875 | Caprazamycin\_biosynthetic\_gene\_cluster | 24 | 33.8008415147 | 109 | 2.4e-23 |
| BAE56595.1 |  | BGC0001123 | Aspirochlorine\_biosynthetic\_gene\_cluster | 25 | 33.5203366059 | 109 | 3.2e-23 |
| AFS60105.1 | PaenT | BGC0000540 | Paenibacillin\_biosynthetic\_gene\_cluster | 25 | 35.1332398317 | 109 | 4.2e-23 |
| CAB60262.1 | MrsT\_protein | BGC0000527 | Mersacidin\_biosynthetic\_gene\_cluster | 25 | 36.5357643759 | 106 | 2.1e-22 |
| CAP20371.1 | ABC\_transporter\_ATP-binding\_membrane\_protein | BGC0000347 | Equibactin\_biosynthetic\_gene\_cluster | 21 | 36.0448807854 | 105 | 4.6e-22 |
| ALK27913.1 | putative\_ABC\_transporter | BGC0001233 | Feglymycin\_biosynthetic\_gene\_cluster | 27 | 23.0715287518 | 104 | 7.8e-22 |
| BAH43767.1 | ABC\_transporter\_ATP\_binding/permease\_protein | BGC0000452 | Tyrocidine\_biosynthetic\_gene\_cluster | 25 | 33.5203366059 | 102 | 3e-21 |
| WP\_043270371.1 | ABC\_transporter\_ATPase | BGC0001188 | Legonaridin\_biosynthetic\_gene\_cluster | 21 | 36.7461430575 | 101 | 8.7e-21 |
| ACU73106.1 | ABC\_transporter\_related | BGC0000501 | Catenulipeptin\_biosynthetic\_gene\_cluster | 27 | 29.873772791 | 101 | 1.1e-20 |
| AAX31526.1 | putative\_ABC\_transporter | BGC0000336 | Daptomycin\_biosynthetic\_gene\_cluster | 26 | 33.5904628331 | 100 | 1.9e-20 |
| CBW74823.1 | Multidrug\_resistance\_ABC\_transporter\_ATP-binding\_and\_permease\_protein | BGC0000571 | Burhizin\_biosynthetic\_gene\_cluster | 26 | 30.2244039271 | 98 | 7.3e-20 |
| ANQ31704.1 | ABC\_transporter | BGC0001395 | Teichomycin\_biosynthetic\_gene\_cluster | 25 | 35.8345021038 | 97 | 9.6e-20 |
| CAX48974.1 | ABC-transporter | BGC0000519 | Labyrinthopeptin\_A1,A3\_/\_labyrinthopeptin\_A2\_biosynthetic\_gene | 25 | 31.4866760168 | 96 | 2.8e-19 |
| AFD30962.1 | CrmT3 | BGC0000966 | Caerulomycin\_A\_biosynthetic\_gene\_cluster | 25 | 28.190743338 | 96 | 3.6e-19 |
| AHW81482.1 | hypothetical\_protein | BGC0000934 | Endophenazines\_biosynthetic\_gene\_cluster | 26 | 32.6788218794 | 95 | 6.2e-19 |
| CAM03502.1 | putative\_ABC\_transporter | BGC0000513 | Erythreapeptin\_biosynthetic\_gene\_cluster | 27 | 22.2300140252 | 94 | 8.1e-19 |
| AAA62651.1 |  | BGC0000504 | Cytolysin\_ClyLl\_/\_cytolysin\_ClyLs\_biosynthetic\_gene\_cluster | 23 | 34.8527349229 | 90 | 1.5e-17 |
| AAK01462.1 | ABC\_transporter | BGC0000412 | Pyochelin\_biosynthetic\_gene\_cluster | 27 | 22.8611500701 | 86 | 2.2e-16 |
| AAG32538.1 | SalT | BGC0000548 | Salivaricin\_A\_biosynthetic\_gene\_cluster | 23 | 36.255259467 | 86 | 3.8e-16 |
| CAB01940.1 | ExpD1 | BGC0000801 | Galactoglucan\_biosynthetic\_gene\_cluster | 26 | 30.8555399719 | 84 | 8.4e-16 |
| ctg1\_orf5 |  | BGC0000321 | Chaetocin\_biosynthetic\_gene\_cluster | 23 | 36.3955119215 | 78 | 6e-14 |
| WP\_003950017.1 | ABC\_transporter\_ATP-binding\_protein | BGC0000546 | SAL-2242\_biosynthetic\_gene\_cluster | 25 | 28.4712482468 | 76 | 2.3e-13 |
| AAC63532.1 | sublancin\_transporter\_protein | BGC0000558 | Sublancin\_168\_biosynthetic\_gene\_cluster | 22 | 35.0631136045 | 75 | 6.7e-13 |
| BAP05577.1 | CalU | BGC0000967 | Calyculin\_biosynthetic\_gene\_cluster | 25 | 29.523141655 | 72 | 5.6e-12 |
| AHF21227.1 | TriC | BGC0000449 | Tridecaptin\_biosynthetic\_gene\_cluster | 23 | 38.7798036466 | 71 | 7.4e-12 |
| BAL72550.1 | lariatin\_biosynthetic\_protein | BGC0000575 | Lariatin\_biosynthetic\_gene\_cluster | 23 | 31.0659186536 | 64 | 1.2e-09 |
| AFD30963.1 | CrmT2 | BGC0000966 | Caerulomycin\_A\_biosynthetic\_gene\_cluster | 22 | 29.3127629734 | 62 | 5.8e-09 |
| ABQ84449.1 | salivaricin\_ABC-transporter | BGC0000624 | Salivaricin\_CRL1328\_alpha\_peptide\_/\_salivaricin\_CRL1328\_beta\_peptide | 22 | 31.556802244 | 60 | 1.7e-08 |
| AGM16415.1 | paenibacterin\_ABC-transporter\_D | BGC0000400 | Paenibacterin\_biosynthetic\_gene\_cluster | 23 | 30.4347826087 | 59 | 3.8e-08 |
| AAG28766.1 | CoaD | BGC0000617 | Coagulin\_biosynthetic\_gene\_cluster | 22 | 30.9256661992 | 58 | 6.4e-08 |
| CAG34716.1 | putative\_ABC\_transporter | BGC0000713 | Ribostamycin\_biosynthetic\_gene\_cluster | 23 | 38.7096774194 | 57 | 1.4e-07 |
| AGL93176.1 | ABC\_transporter\_ATP-binding\_protein | BGC0000560 | Subtilomycin\_biosynthetic\_gene\_cluster | 23 | 28.190743338 | 56 | 3.2e-07 |
| AHZ20760.1 | ABC\_transporter\_transmembrane\_domain/ATP-binding\_domain\_protein | BGC0000369 | Hassallidins\_biosynthetic\_gene\_cluster | 22 | 36.5357643759 | 55 | 5.5e-07 |
